# Hepatic reference gene selection in adult and juvenile female Atlantic salmon at normal and elevated temperatures

**DOI:** 10.1186/1756-0500-5-21

**Published:** 2012-01-10

**Authors:** Kelli C Anderson, Abigail Elizur

**Affiliations:** 1 Faculty of Science, Health and Education, University of the Sunshine Coast, Locked Bag 4, Maroochydore DC, Queensland 4558, Australia; 2 Australian Seafood Cooperative Research Centre, Box 26, Mark Oliphant Building, Science 10 Park Adelaide, Laffer Drive, Bedford Park, Adelaide SA, 5042, Australia

## Abstract

**Background:**

The use of quantitative real-time polymerase chain reaction (qPCR) has become widespread due to its specificity, sensitivity and apparent ease of use. However, experimental error can be introduced at many stages during sample processing and analysis, and for this reason qPCR data are often normalised to an internal reference gene. The present study used three freely available algorithms (GeNorm, NormFinder and BestKeeper) to assess the stability of hepatically expressed candidate reference genes (Hprt1, Tbp, Ef1α and β-tubulin) in two experiments. In the first, female Atlantic salmon (*Salmo salar*) broodstock of different ages were reared at either 14 or 22°C for an entire reproductive season, therefore a reference gene that does not respond to thermal challenge or reproductive condition was sought. In the second, estrogen treated juvenile salmon were maintained at the same temperatures for 14 days and a reference gene that does not respond to temperature or estrogen was required. Additionally, we performed independent statistic analysis to validate the outputs obtained from the program based analysis.

**Results:**

Based on the independent statistical analysis performed the stability of the genes tested was Tbp > Ef1α > Hprt1 > β-tubulin for the temperature/reproductive development experiment and Ef1α > Hprt1 > Tbp for the estrogen administration experiment (β-tubulin was not analysed). Results from the algorithms tested were quite ambiguous for both experiments; however all programs consistently identified the least stable candidate gene. BestKeeper provided rankings that were consistent with the independent analysis for both experiments. When an inappropriate candidate reference gene was used to normalise the expression of a hepatically expressed target gene, the ability to detect treatment-dependent changes in target gene expression was lost for multiple groups in both experiments.

**Conclusions:**

We have highlighted the need to independently validate the results of reference gene selection programs. In addition, we have provided a reference point for those wishing to study the effects of thermal challenge and/or hormonal treatment on gene stability in Atlantic salmon and other teleost species.

## Background

The use of quantitative real-time polymerase chain reaction (qPCR) has become widespread due to its specificity, sensitivity, broad dynamic range, cost effectiveness, high throughput capability and the need to measure exact levels of gene transcription [1,2]. Despite its apparent ease of use, there are many stages in between initial sampling and performance of the qPCR that can introduce variability, and essentially affect the quality and reliability of the data produced.

There are a few common standardisation techniques that are used to compensate for introduced variability; for example, data can be normalised to sample size (tissue weight or number of cells), quantity of RNA extracted, or a stably expressed reference or 'housekeeping' gene [1]. However, there are potentially significant drawbacks associated with each of these methods. RNA extractions are routinely carried out on tissue samples that may contain various cell types. For this reason, ensuring that the cellular make-up of dissected tissue is consistent between animals of different disease or developmental state can be quite difficult [1], and the specific contribution of each cell type to the total amount of RNA extracted could be disproportionate between samples. Subsequently, qPCR results acquired may reflect changes in cell composition and not a response to experimental conditions. While ensuring that the input amount of total RNA is the same between samples for cDNA synthesis is essential, this should not be used as a complete standardisation strategy. Standard RNA quantification methods mostly measure the ribosomal RNA (rRNA) fraction that can account for ~80% of total RNA. Therefore, this method relies on the assumption that the ratio of messenger RNA (mRNA) to rRNA does not change as a result of the experimental treatment or condition [3,4]. However, previous studies have demonstrated that stability of the rRNA fraction cannot be taken for granted [5,6]. Additionally, this method of standardisation does not control for pipetting error during cDNA synthesis, inhibitory factors contained in the tissue, or error introduced during the reverse transcription and qPCR phases of sample processing and analysis respectively [1]. Normalisation to one or several internal control (reference) genes is by far the most common method used to manage technical or other variation when estimating gene expression levels. Its inclusion in qPCR studies is preferred over other methods of normalisation since the reference mRNA template is present at all stages of processing and analysis, and will therefore reflect the cumulative change in sample dynamics [7]. However, inappropriate use of a single or multiple reference genes for normalisation can limit one's ability to detect small changes in mRNA abundance, or alter the fundamental findings and conclusions of a study [8].

In the past, many studies have assumed the stability of a small group of 'classic' reference genes without proper validation [4]. However, guidelines for reference gene selection are becoming more stringent, and there are now various prerequisites that a candidate reference gene must fulfill before it can be considered appropriate for normalising experimental error. For instance, the reference gene transcript level should not change as a result of experimental treatment [4], it should be expressed at a level that is similar to that of the target gene [1,9] and amplification should be RNA specific with the absence of pseudogenes and contaminating genomic DNA (gDNA) [10]. In fish species, it is now apparent that the expression of many commonly used reference genes may vary on the basis of gender [11], tissue type [12], developmental stage [13] and experimental conditions such as type and length of exposure to exogenous chemicals [14]. Therefore, indiscriminate use of reference genes without stringent testing and validation could lead to incorrect expression profiling and interpretation of results [8,11].

For many studies, a question remains as to what is the best way to determine whether candidate reference genes are appropriate for normalising non-biological sample variation. At the present time, there are three popular algorithms which have been specifically designed to determine the most suitable reference gene or combination of genes from a panel of candidates, namely BestKeeper [7], GeNorm [4] and NormFinder [15]. Recently it has been demonstrated that discrepancy can sometimes occur between results obtained from these software packages due to differences between algorithms, and it has been suggested that additional external evaluation is necessary to independently confirm the validity of genes selected by computer programs [16-18].

Salmonids are the most widely studied group of teleosts. However, studies investigating the usefulness of reference genes in qPCR have mainly focused on immature salmon [14,19,20], and no data are available for the expression of candidate reference genes for adult fish over an entire reproductive season. In addition to this, there are fundamental knowledge gaps concerning whether the age of an adult fish, or rearing temperatures outside of the optimum range modulate the expression of reference genes as demonstrated for other target genes during reproductive development [21]. In order to be able to assess the effects of temperature on reproduction, a reference gene is required whose expression is stable throughout reproductive development and across temperature ranges. In the present study, we have assessed the expression stability of elongation factor 1 alpha (Ef1α), hypoxanthine phosphoribosyltransferase 1 (Hprt1), TATA-box-binding protein (Tbp) and β-tubulin, all of which are routinely used reference genes in the field of teleost reproductive physiology. Gene expression was measured in hepatic tissue from 2+ (maiden) and 3+ (repeat) year old female Atlantic salmon reared at either 14 or 22°C during a reproductive season. Reference gene stability was then determined using the freely available BestKeeper, GeNorm and NormFinder algorithms, and methods recommended elsewhere in the literature [17]. Then using the same method, we determined whether the same reference gene candidates (except β-tubulin) were suitable normalising genes in a study where juvenile Atlantic salmon were given either a blank or 17β-estradiol (E_2_) implant, and maintained at either 14 or 22°C for 14 days. Studies such as these could become even more critical in years to come as wild and farmed fish species are affected by climate change, and we explore novel methods such as hormonal therapy to improve reproductive performance under thermal challenge.

## Results

### qPCR assay validations

The efficiency and R^2 ^value for all primer pairs were above 0.96 and 0.95 respectively (Table 1). For each reaction a single amplicon was produced as determined via melt curve analysis and agarose gel electrophoresis (data not shown); amplicon identity was confirmed through sequencing. No detectable qPCR product was produced for no-template controls and negative reverse transcription controls which confirms the absence of contamination by gDNA or other sources.

**Table 1:** qPCR primers used to amplify fragments of candidate reference genes

Gene	Primer	Sequence (5'→3')	Amplicon size	E*	R^2^	GenBank #
Hprt1	Hprt1F1	GAT GAT GAG CAG GGA TAT GAC	165 bp	0.963	0.999	BT043501
	Hprt1R1	GCA GAG AGC CAC GAT ATG G				

Tbp	TbpF1	TCC CCA ACC TGT GAC GAA CA	117 bp	0.981	0.958	BT059217
	TbpR1	GTC TGT CCT GAG CCC CCT GA				

Ef1α	Ef1αF2	GCA CCA CGA GAC CCT GGA AT	94 bp	0.969	0.997	AF321836
	Ef1αR2	CAC GTT GCC ACG ACG GAT AT				

β-tubulin	βTubF1	CCG TGC TTG TCG ACT TGG AG	144 bp	0.975	0.998	DQ367888
	βTubR2	CAG CGC CCT CTG TGT AGT GG				

*E = efficiency, *bp *= base pairs

### Stability analysis of candidate reference genes

**Figure 1:**
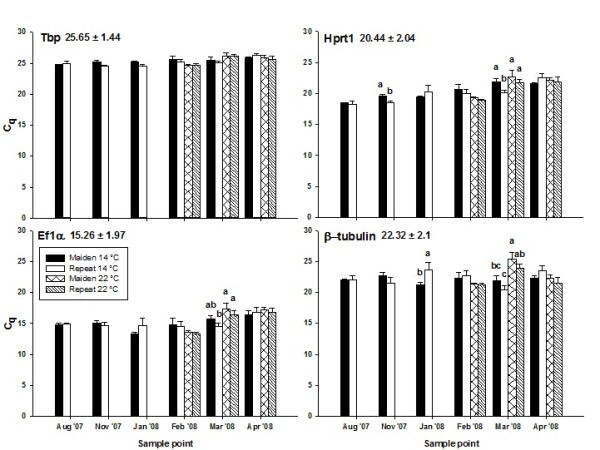
**Abundance of hepatic mRNA transcripts for Tbp, Hprt1 and Ef1α and β-tubulin in female maiden Atlantic salmon reared during the reproductive season**. The average quantification cycle (C_q_) of all groups analysed, and the standard error is shown at the top left of the graph. Bars show the average C_q _(+SEM) for each group of fish (n = 7). Different superscripts denote statistical significance between groups at a given sample point at *p *≤ 0.05.

In the adult broodstock experiment, Ef1α consistently had the highest transcript abundance, Hprt1 and β-tubulin had intermediate transcript abundance, and Tbp had the lowest (Figure 1, raw data are available at http://doi.pangaea.de/10.1594/PANGAEA.772212) Additional file 1: Table S1. When considering the standard error of the average quantification cycle (C_q_) for all groups of fish studied, Tbp had the lowest transcript abundance variance followed by Ef1α, Hprt1 and β-tubulin (Figure 1). When Kruskal-Wallis analysis coupled with Bonferroni's correction was performed on a month-by-month basis, no significant differences in transcript abundance were found for Tbp during the reproductive season (Figure 1). For Ef1α, significant differences in transcript abundance were found for two sets of statistical comparison, for Hprt1 four sets of comparison were statistically significant and for β-tubulin five sets of comparison were statistically significant (Figure 1). When Kendall's tau correlation analysis was performed between C_q _value and sample point, significant C_q_-time relationships were found for all genes at *p *≤ 0.01 except β-tubulin. For Tbp, sample point accounted for 7.2% of C_q _variation, for Ef1α time of sampling accounted for 9.3% of C_q _and for Hprt1 time accounted for 29.37% of C_q _variation.

**Figure 2:**
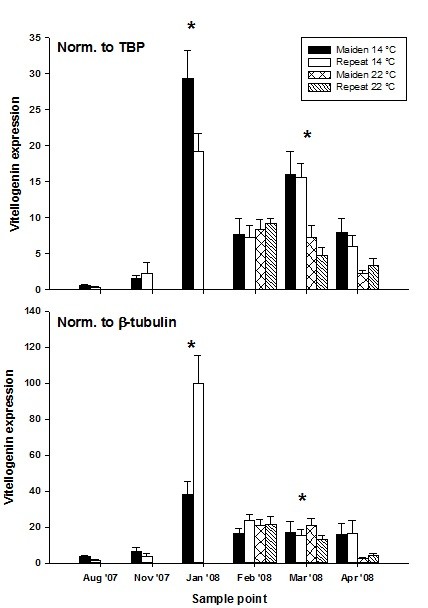
**Vitellogenin gene expression normalised to either Tbp or β-tubulin in female maiden Atlantic salmon reared at either 14 or 22°C and repeat salmon reared during the reproductive season**. The mean (+ SEM) gene expression levels for each group of fish (n = 7) are displayed. The asterisk is placed above sample points where different target gene expression results were obtained after normalization.

The BestKeeper algorithm ranked Tbp as the most appropriate reference gene while GeNorm ranked Tbp and Hprt1 as the most suitable pair after the step-wise elimination of the least stable genes (Table 2). This partly agreed with the results from NormFinder where Tbp was ranked third and Hprt1 was ranked first. Hprt1 was given a rank of three by BestKeeper which was not in agreement with any other algorithm. Ef1α was given a rank of two by BestKeeper and NormFinder and three by GeNorm. However, all three algorithms were in agreement when β-tubulin was assigned a rank of four, and was named as the least appropriate candidate gene for use in qPCR normalisation. When the hepatic expression of the target gene vitellogenin (egg yolk protein) was normalised using β-tubulin, then compared to the data normalised by Tbp, the ability to detect treatment-dependent changes in target gene expression was lost during January and March (Figure 2, raw data are available at http://doi.pangaea.de/10.1594/PANGAEA.772212) Additional file 2: Table S2.

**Table 2:** Ranking of candidate reference genes

	Adult experiment			Juvenile experiment		
**Gene name**	**BK (corr.)**	**NF (stab. value)**	GN*(M)	**BK (corr.)**	**NF (stab. value)**	GN*(M)

Tbp	1 (0.965)	3 (0.106)	1 (0.796)	3 (0.852)	3 (0.154)	3 (0.614)

Ef1α	2 (0.925)	2 (0.09)	3 (0.878)	1 (0.936)	2 (0.146)	1 (0.537)

Hprt1	3 (0.923)	1 (0.081)	1 (0.752)	2 (0.912)	1 (0.065)	1 (0.568)

β-tubulin	4 (0.873)	4 (0.122)	4 (0.913)	-	-	-

BestKeeper (BK), Norm Finder (NF) and GeNorm (GN); (corr.) = correlation between candidate gene and BestKeeper index, (stab. value) = stability value calculated by NormFinder and (M) = stability value calculated by GeNorm with all genes included. GN ranks were determined through step-wise elimination of the least stable gene. For NF and GN a lower value is indicative of a more stable gene. For BK a higher value is indicative of a more stable gene. - β-tubulin was not analysed in the juvenile study. * GeNorm selects the best pair of candidate genes, not a single gene

**Figure 3:**
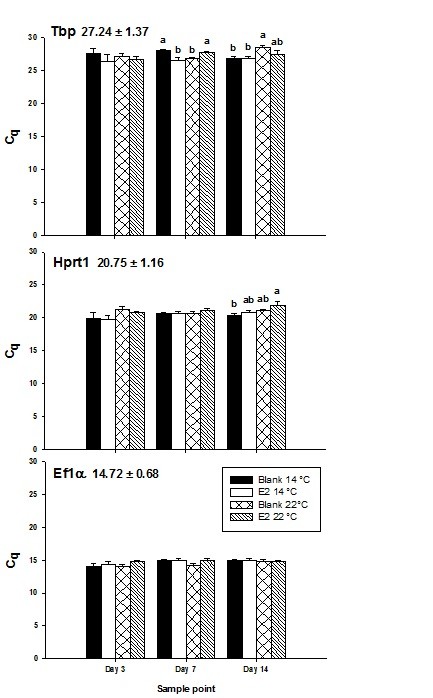
**Abundance of hepatic mRNA transcripts for Tbp, Hprt1 and Ef1α in juvenile Atlantic salmon given a blank silastic implant**. The average quantification cycle (C_q_) of all groups analysed, and the standard error is shown at the top left of the graph. Bars show the average C_q _(+ SEM) for each group of fish (n = 7). Different superscripts denote statistical significance between groups at a given sample point at *p *≤ 0.05.

In the juvenile salmon experiment, Ef1α had the highest transcript abundance followed by Hprt1 then Tbp (Figure 3, raw data are available at http://doi.pangaea.de/10.1594/PANGAEA.772213) Additional file 3: Table S3. This order is consistent with results from the adult experiment and in fact, the average C_q _values for each gene between the different studies were very similar. Ef1α had the lowest transcript abundance variance as demonstrated by the low standard error for this gene, Hprt1 had an intermediate level of variance and Tbp had the highest (Figure 3). The standard error for the three genes examined in this experiment ranged between 0.68 and 1.4 cycles which is lower than 1.44 and 2.04 cycles that was observed for the same genes (not including β-tubulin) in the adult experiment. Kruskal-Wallis analysis followed by Bonferroni's correction revealed that no significant differences in C_q _existed between experimental groups within a given sample point for EF1α (Figure 3). The same could not be said for Hprt1 where one significantly different comparison was found or for Tbp where six sets of comparisons were statistically significant. Kendall's tau correlation analysis revealed a small but significant relationship between Ef1α C_q _and sample point, where sample point accounted for 3.61% of C_q _variation (*p *≤ 0.05). No significant relationship between time and expression of Tbp or Hprt1 was found.

Unlike the adult experiment where two out of three programs listed Tbp as an acceptable reference gene, all three algorithms ranked Tbp as the least stable reference gene candidate in the juvenile experiment (Table 2). GeNorm selected Ef1α and Hprt1 as the most appropriate pair of candidates, BestKeeper selected Ef1α and NormFinder selected Hprt1 as the best candidate. It is interesting that NormFinder listed Hprt1 as the most stable single gene, though listed Ef1α and Tbp as the best combination of two genes to use for target gene normalisation. Similarly, addition of the least stably expressed gene (Tbp) into a panel of Hprt1 and Ef1α made a significant contribution (0.24) to the normalisation factor calculated by GeNorm despite its lower ranking. Based on the results from GeNorm, all three reference genes should be used for accurate normalisation of qPCR data.

## Discussion

We have reported on the stability of candidate reference genes for the entire reproductive season of maiden and repeat spawning female Atlantic salmon reared under cool (14°C) and warm (22°C) conditions in Tasmania. Statistical analysis of Tbp C_q _value revealed that no significant differences were present between experimental groups of fish within a given sampling point for the entire reproductive season. Tbp also had the lowest transcript abundance variance over the entire eight month period which suggests that it is the most stably expressed gene of the candidate panel tested. However, correlation analysis revealed that 7.2% of the variation in transcript abundance could be accounted for by sampling point. This may indicate that Tbp gene expression is down-regulated (has a higher C_q_) to some extent over time because it is linked to reproductive status. For this reason, a degree of caution should be exercised when directly comparing the normalised expression levels of target genes from fish sampled in different months, particularly between the start and end of the reproductive season (i.e. August'07 versus April'08) as normalisation could introduce some error.

Two statistically significant comparisons were found for the C_q _value of Ef1α during March; in November and March a total of four significant comparisons were found for Hprt1 and five were found for β-tubulin. Therefore, it is not surprising that EF1α had the second lowest level of C_q _variance followed by Hprt1 then β-tubulin. The presence of statistically significant differences in C_q _value indicates that these genes may not be suitable candidates for target gene normalisation during certain months of the year. In fact, normalisation of a hepatically expressed target gene (vitellogenin) to β-tubulin resulted in a loss of the ability to detect treatment-dependent changes in target gene expression during January and March when compared to target gene expression data normalised by Tbp (Figure 2). These results confirm the work of previous authors where inappropriate use of a reference gene significantly altered the interpretation of qPCR results [8,22].

In a similar fashion to Tbp, a significant correlation between sample point and C_q _was found for Ef1α that accounted for 9.3% of C_q _variation. Again, it may be at the discretion of the researcher to decide whether comparisons of qPCR data are of critical importance between months at the start and end of the reproductive season, or whether within month assessment of gene expression levels will suffice. However, the strongest relationship between sample point and transcript abundance was found for Hprt1 as ~30% of C_q _variance could be attributed to the month of sampling. The apparent connection between gene expression and developmental state, the number of statistically significant comparisons found and high standard error compared to other candidate genes made Hprt1 a poor choice for qPCR data normalisation in the present study. Even though no significant correlation was found between β-tubulin and sample point, the use of a β-tubulin to normalise target gene expression variance resulted in experimental bias due to a combination of its high standard error and high within-month C_q _variation (Figure 2). Based on the statistical analysis performed, the usefulness of candidate reference genes for normalisation is as follows: Tbp > Ef1α > Hprt1 > β-tubulin.

The BestKeeper algorithm provided rankings that were in agreement with the ranking obtained (above) via independent statistical analysis. GeNorm also selected Tbp as an ideal reference gene, however this gene was chosen in combination with Hprt1 which showed the largest relationship between C_q _and sample point, and four statistically significant comparisons. However, GeNorm successfully identified β-tubulin as the least stably expressed candidate gene. NormFinder also ranked β-tubulin as the least suitable gene for target gene normalisation. However, unlike the other algorithms, NormFinder assigned a rank of three to Tbp which is surprising given the lower standard error, within month variance and C_q_-time correlation of Tbp compared to the rest of the candidate panel. However, ranking of the remaining genes between algorithms was ambiguous which indicates that the output of such programs should not be used complacently.

Disagreement between programs concerning the suitability of candidate reference genes presumably occurred as a result of the different statistical principles and assumptions underlying each algorithm. For example, the BestKeeper program assumes that if candidate reference genes are stably expressed, then gene expression levels should be highly correlated. Thus, pair-wise correlations for every possible gene pair combination are performed, and the geometric means of highly correlated genes are used to calculate an index. The level of correlation between each individual gene and the index is then determined, and a higher coefficient of correlation is indicative of a more stable gene. Unlike BestKeeper, GeNorm uses relative gene expression levels (calculated using the ΔCq method) and not raw Cq values to calculate the average pair-wise variation between candidate genes. A stability value (M) calculated as a result of this analysis can then be used to eliminate the least stable gene in a step-wise fashion until only the two genes remain. Lastly, NormFinder allows the user to define experimental treatments through the use of group identifiers which is a feature unique to this software. As a result, NormFinder takes into account both inter- and intra-group variability when using relative gene expression levels to calculate a stability value then subsequently rank candidate genes. Based on our data, it appears that all three reference gene selection programs are useful for eliminating the least stably expressed gene from a panel of candidates. For this reason external validation of program outputs is warranted to account for conflict between algorithms, program assumptions and limitations when complex experimental designs are employed.

In addition to the adult experiment, we also determined the suitability of the same candidate reference genes (except β-tubulin) for use in a study where juvenile salmon were given a blank or E_2 _pellet and reared at either 14°C or 22°C for 14 days. In the juvenile study, the gene expression of Ef1α did not significantly change as a result of experimental treatment within each sampling point. However, a significant C_q_- sample point correlation was found for this gene that accounted for 3.61% of C_q _variance. While this is a statistically significant relationship (*p *≤ 0.05), it is probably not of major concern due to its magnitude. One significant comparison was detected for Hprt1 gene expression at day 14, and a total of six sets of comparison were significant for Tbp. Ef1α also had the lowest transcript abundance variance, followed by Hprt1 then Tbp. This strongly suggests that stability of candidate reference genes in this experiment is as follows: Ef1α > Hprt1 > Tbp.

All three reference gene programs were in agreement when assigning a rank of three to Tbp which was the same result achieved through external statistical analysis (above). Through this analysis it has become apparent that freely available algorithms are consistently able to identify the least stable gene from a panel of candidates. BestKeeper found Ef1α to be the most stable candidate followed by Hprt1 while the opposite was found using NormFinder. In a similar fashion to the adult experiment, the BestKeeper algorithm gave the same rankings to the reference gene candidates as independent statistical analysis and therefore has been the most reliable algorithm in our studies. GeNorm selected Ef1α and Hprt1 as the most suitable pair of candidates; although the addition of Tbp to the gene panel significantly improved overall stability value (by 0.24) despite its apparent lower stability. When the expression of a hepatically expressed gene was normalised to Tbp alone or in combination with Hprt1 and Ef1α, significantly different results were achieved for two groups of fish (data not shown). Additionally, variance in the target gene expression data, and therefore noise, was reduced when all three genes were used for normalisation instead of Tbp alone (data not shown).

In a broad sense, our data agree with previous studies that outline the need to validate reference genes on an experiment-to-experiment basis [11,14]. For example, in the adult experiment there is strong evidence to suggest that Tbp was the best candidate for target gene normalisation, while in the juvenile experiment Tbp was ranked third but may still prove useful as a reference gene in combination with other candidates. Based on our data, we recommend Tbp and Ef1α as a starting point when selecting candidate reference gene for research using female Atlantic salmon broodstock where hepatic gene expression will be measured during reproductive development. Furthermore, Ef1α and Hprt1 appear to be quite stable in juvenile fish treated with E_2 _under thermal challenge. As a final point, all three algorithms correctly identified the least stable candidates in both of our experiments and can therefore prove useful to initially screen data and eliminate the most undesirable genes.

## Conclusion

In recent years it has become clear that no single gene is stably expressed under all experimental conditions for any given tissue or species. Our study further highlights the need to evaluate reference gene stability separately for every experiment as it is likely that no one gene will be consistently stable across experiments. Freely available stability assessment programs are user friendly and can provide valuable information in gene expression studies. However, we recommend that independent statistical validation be carried out as an additional safe guard against inappropriate adjustment of target gene expression. Furthermore, full justification for the reference genes selected should be provided in any publications containing relative qPCR data.

## Methods

### Sampling

For the adult experiment, maiden and repeat cultured adult females were held at the Salmon Enterprises of Tasmania (SALTAS) Wayatinah Hatchery (Tasmania, Australia) at ambient temperature and photoperiod in either 200 (maidens) or 50 (repeats) m^3 ^circular tanks at stocking densities of 12-18, and 24-36 kg m^-3 ^for maidens and repeats, respectively until early January 2008. In January, fish were transferred to temperature-controlled 4 m^3 ^tanks (14 fish per tank) under simulated ambient photoperiod according to the following experimental groups (28 fish per group): maidens reared at 14°C, repeats reared at 14°C, maidens reared at 22°C and repeats reared at 22°C. Fish were not fed from the time of transfer to the temperature controlled systems in January which is consistent with hatchery practice for management of this experimental stock of fish. All fish were maintained at the nominated temperature (14 or 22°C) until early April when all fish were exposed to a temperature ramp down over 11 days to 8°C to induce final oocyte maturation and ovulation as described in King and Pankhurst [23].

Fish from both maiden and repeat groups were sampled on the 31st August and 2nd November 2007, and 7th January 2008 and after introduction to the controlled temperature regimes on the 14th February 2008, 28th March and 25th April. For sampling, fish were netted from the holding tanks, terminally anaesthetised in Aqui-S ™ (Crop & Food, New Zealand) and sections of liver were transferred to 1-2 mL of RNA Later ™ (Qiagen, Germany) to stabilise mRNA for later measurement of gene expression. Samples were held overnight at 4°C, then stored at -20°C. This research activity was undertaken with approval from the Animal Ethics Committees of the University of the Sunshine Coast and Griffith University (approval numbers AN/A/07/35 and EAS/02/07/AEC respectively).

For the juvenile experiment, 84 juvenile female Atlantic salmon (217.2 g ± 4.68 g) were housed at the SALTAS, in 4 separate 1000 L tanks (21 fish per tank) with independent recirculating fresh water for each temperature treatment. At day 0, all fish were anesthetised with Aqui-S™ (25 ppm), weighed, implanted with a blank or E_2_-containing pellet (10 mg.kg^-1^) and placed in thermo-regulated tanks in one of four experimental groups: E_2 _pellet at 14°C, E_2 _pellet at 22°C, blank silastic pellet at 14°C and blank silastic pellet at 22°C (n = 21 per group). At 3, 7 and 14 days post implantation, 7 fish were sacrificed from each group using a lethal dose of Aqui-S™ (50 ppm). Liver sections were dissected and stored as described above. This experiment was conducted under approval from the Animal Ethics Committee of the University of the Sunshine Coast (approval number AN/A/07/35).

### RNA isolation and cDNA synthesis

Total RNA was isolated from 15 mg of hepatic tissue using the Illustra RNAspin Mini kit (GE Healthcare) according to the manufacturer's protocol. RNA yield and 260/280 purity ratio were determined using the NanoDrop 2000 (Thermo Scientific). An RNA integrity number (RIN) was determined for a random sample of hepatic RNA using a 2100 Bioanalyzer (Agilent) to establish RNA quality. All RNA was stored at -80°C until use.

Four hundred nanograms of liver-derived RNA were used to synthesise cDNA for use in qPCR using the QuantiTect^® ^reverse transcription kit (Qiagen). This kit includes a DNA elimination step to remove potential contamination of PCRs by genomic DNA. Following synthesis, cDNA was stored at -20°C until use.

### Primer design and standard curve construction

Gene specific primers for Hprt1, Ef1α, β-tubulin and Tbp were designed from species-specific mRNA sequences (freely available on GenBank) using Primer3 software (http://frodo.wi.mit.edu/primer3/, Table 1) by Pankhurst et al., (2011). All primers were designed to have an optimum annealing temperature of 60°C and were supplied by GeneWorks (Australia). Validation curves containing at least five points were carried out in triplicate using serially diluted cDNA as the template. Following amplification, the size of all qPCR products was determined by running 4 μl of the product on a 2% agarose gel; gene identity was then confirmed through sequencing. Reaction efficiencies (Table 1) were automatically calculated by Rotor-gene software version 1.7.87 using the equation: E =[10^(-1/M)^]-1, where E is equal to efficiency and M is equal to slope.

### qPCR cycling conditions

qPCRs were conducted on a Rotor-gene 6000 series thermal cycler (Qiagen) using the following reaction mix: 5 μl Platinum^® ^SYBR^® ^Green qPCR SuperMix-UDG (Invitrogen), 200 nM each primer, 3.6 μl PCR grade water and 1 μl cDNA template (total 10 μl). Cycling conditions were as follows: 50°C for 2 min; 95°C for 2 min; 40 cycles of 95°C for 15 sec; 60°C for 15 sec, and 72°C for 20 sec (acquiring). At the end of cycle 40, a melt curve analysis was performed to confirm amplification of a single product as follows: 90 sec preconditioning step at 72°C, followed by a temperature gradient up to 95°C at 1°C per 5 sec. For every gene analysed no-template controls (NTCs) were included to detect possible contamination; negative reverse transcriptase controls were also analysed to detect contaminating DNA if present.

### Candidate reference gene analysis

Raw C_q _values were transformed to relative expression levels using the 'ΔC_q_' method and the equation Q = E^(minCq - sampleCq) ^where Q = relative quantities, E = efficiency (+ 1) and minC_q _refers to the C_q _of the sample with the highest expression level (lowest C_q_) in the data set for any given gene. Input for GeNorm and NormFinder were data transformed to relative expression levels using the ΔCq method, stability was assessed according to the program's instructions. Sub-group identifiers were included for NormFinder analysis. For BestKeeper, data were entered without modification as raw C_q _according to the program's instructions.

Previous authors have recommended that statistical differences in qPCR be detected non-parametrically [17,24]. Therefore, statistical differences between groups on a monthly basis were analysed using the Kruskal-Wallis test coupled with Bonferroni's Correction to reduce the risk of type 1 error using SPSS (version 17.0). The P value for all analyses was initially set at 0.05 before adjustment by Bonferroni's correction. Kendall's tau non-parametric correlation analysis was performed to assess whether a significant relationship existed between raw C_q _value and sampling point for each gene in both experiments. The R^2 ^method was used for interpretation of the correlation results where the correlation coefficient is squared, then multiplied by 100 to give the percent of variation in C_q _accounted for by time. Target gene (vitellogenin) expression was calculated using Relative Expression Software Tool (REST^©^) [25].

### Availability of supporting data

The data sets supporting the results of this article are available in the PANGAEA^® ^repository (http://doi.pangaea.de/10.1594/PANGAEA.772212 and http://doi.pangaea.de/10.1594/PANGAEA.772213 for the adult and juvenile experiments respectively).

## Competing interests

The authors declare that they have no competing interests.

## Authors' contributions

KA took part in sampling, performed all laboratory work/data analysis and drafted the manuscript. AE acquired funding, took part in sampling, critically reviewed the manuscript and provided principal supervision for KA.

## Supplementary Material

Additional file 1 **Table S1**. Abundance of hepatic mRNA transcripts for Tbp, Hprt1 and Ef1α and β-tubulin in female maiden and repeat Atlantic salmon reared at either 14 or 22°C during the reproductive season. Click here for file

Additional file 2 **Table S2**. Vitellogenin gene expression normalised to either Tbp or β-tubulin in female maiden and repeat Atlantic salmon reared at either 14 or 22°C during the reproductive season. Click here for file

Addtional file 3 **Table S3**. Abundance of hepatic mRNA transcripts for Tbp, Hprt1 and Ef1α in juvenile Atlantic salmon given a blank or E_2 _silastic implant at 14 or 22°C. Click here for file
