# Supplementary material for: Hepatic reference gene selection in adult and juvenile female Atlantic salmon at normal and elevated temperatures
Source: BMC Res Notes. 2012 Jan 10;5:21. doi: 10.1186/1756-0500-5-21 (PMC3392733; doi:10.1186/1756-0500-5-21)
Supplement: Additional file 1 — Table S1. Abundance of hepatic mRNA transcripts for Tbp, Hprt1 and Ef1α and β-tubulin in female maiden and repeat Atlantic salmon reared at either 14 or 22°C during the reproductive season. [file 1756-0500-5-21-S1.DOCX]

**Table 1 Abundance of hepatic mRNA transcripts for Tbp, Hprt1 and Ef1α and β-tubulin in female maiden and repeat Atlantic salmon reared at either 14 or 22 °C during the reproductive season**

| **Experimental group** | **Sample point** | **Mean candidate reference gene C_q_ and SEM** | | | | | | | |
| --- | --- | --- | --- | --- | --- | --- | --- | --- | --- |
|  |  | **Tbp** | | **Hprt1** | | **Ef1α** | | **β-Tubulin** | |
| Maiden 14 °C | August 2007 | 24.77 | 0.09 | 18.45 | 0.06 | 14.84 | 0.15 | 22.04 | 0.13 |
| Repeat 14 °C | August 2007 | 24.99 | 0.39 | 18.29 | 0.45 | 14.88 | 0.25 | 22.03 | 0.61 |
| Maiden 14 °C | November 2007 | 25.20 | 0.30 | 19.58 | 0.27 | 15.08 | 0.36 | 22.72 | 0.57 |
| Repeat 14 °C | November 2007 | 24.51 | 0.20 | 18.53 | 0.23 | 14.61 | 0.55 | 21.44 | 0.94 |
| Maiden 14 °C | January 2008 | 25.19 | 0.19 | 19.39 | 0.23 | 13.31 | 0.29 | 21.29 | 0.29 |
| Repeat 14 °C | January 2008 | 24.56 | 0.20 | 20.23 | 1.10 | 14.70 | 1.13 | 23.69 | 1.13 |
| Maiden 14 °C | February 2008 | 25.55 | 0.55 | 20.67 | 0.83 | 14.81 | 0.99 | 22.30 | 0.91 |
| Repeat 14 °C | February 2008 | 25.21 | 0.43 | 20.01 | 0.62 | 14.49 | 0.81 | 22.75 | 0.73 |
| Maiden 22 °C | February 2008 | 24.66 | 0.16 | 19.30 | 0.18 | 13.54 | 0.26 | 21.35 | 0.18 |
| Repeat 22 °C | February 2008 | 24.73 | 0.16 | 18.88 | 0.20 | 13.28 | 0.33 | 21.17 | 0.32 |
| Maiden 14 °C | March 2008 | 26.50 | 0.50 | 21.92 | 0.55 | 15.72 | 0.57 | 21.95 | 0.78 |
| Repeat 14 °C | March 2008 | 25.03 | 0.56 | 20.11 | 0.44 | 14.84 | 0.55 | 20.37 | 0.71 |
| Maiden 22 °C | March 2008 | 27.01 | 0.69 | 22.73 | 1.11 | 17.40 | 0.93 | 25.37 | 1.03 |
| Repeat 22 °C | March 2008 | 26.92 | 0.52 | 21.68 | 0.60 | 16.46 | 0.58 | 23.90 | 0.72 |
| Maiden 14 °C | April 2008 | 25.87 | 0.11 | 21.66 | 0.14 | 16.38 | 0.17 | 22.23 | 0.50 |
| Repeat 14 °C | April 2008 | 26.49 | 0.30 | 22.57 | 0.60 | 16.86 | 0.71 | 23.46 | 0.83 |
| Maiden 22 °C | April 2008 | 26.86 | 0.35 | 22.11 | 0.50 | 17.16 | 0.47 | 22.26 | 0.56 |
| Repeat 22 °C | April 2008 | 25.84 | 0.59 | 21.91 | 0.76 | 16.83 | 0.71 | 21.53 | 0.85 |
