# Supplementary material for: Hepatic reference gene selection in adult and juvenile female Atlantic salmon at normal and elevated temperatures
Source: BMC Res Notes. 2012 Jan 10;5:21. doi: 10.1186/1756-0500-5-21 (PMC3392733; doi:10.1186/1756-0500-5-21)
Supplement: Additional file 2 — Table S2. Vitellogenin gene expression normalised to either Tbp or β-tubulin in female maiden and repeat Atlantic salmon reared at either 14 or 22°C during the reproductive season. [file 1756-0500-5-21-S2.DOCX]

**Table 2 Vitellogenin gene expression normalised to either Tbp or β-tubulin in female maiden and repeat Atlantic salmon reared at either 14 or 22 °C during the reproductive season**

| **Experimental group** | **Sample point** | **Mean normalised Vtg gene expression and SEM** | | | |
| --- | --- | --- | --- | --- | --- |
|  |  | **Tbp** | | **β-tubulin** | |
| Maiden 14 °C | August 2007 | 0.60 | 0.18 | 3.52 | 0.96 |
| Repeat 14 °C | August 2007 | 0.27 | 0.19 | 1.36 | 0.84 |
| Maiden 14 °C | November 2007 | 1.61 | 0.36 | 6.61 | 1.87 |
| Repeat 14 °C | November 2007 | 2.25 | 1.58 | 1.95 | 1.2 |
| Maiden 14 °C | January 2008 | 29.38 | 3.94 | 38.27 | 7.20 |
| Repeat 14 °C | January 2008 | 19.28 | 2.45 | 99.62 | 15.72 |
| Maiden 14 °C | February 2008 | 7.65 | 2.33 | 12.51 | 2.53 |
| Repeat 14 °C | February 2008 | 7.32 | 1.61 | 23.65 | 3.19 |
| Maiden 22 °C | February 2008 | 8.42 | 1.31 | 21.20 | 3.23 |
| Repeat 22 °C | February 2008 | 9.18 | 0.72 | 21.75 | 4.49 |
| Maiden 14 °C | March 2008 | 16.02 | 3.15 | 17.16 | 5.95 |
| Repeat 14 °C | March 2008 | 15.63 | 1.89 | 15.15 | 3.30 |
| Maiden 22 °C | March 2008 | 7.33 | 1.65 | 21.21 | 3.47 |
| Repeat 22 °C | March 2008 | 4.71 | 1.20 | 13.41 | 2.18 |
| Maiden 14 °C | April 2008 | 7.95 | 2.00 | 15.87 | 6.14 |
| Repeat 14 °C | April 2008 | 6.00 | 1.61 | 16.48 | 7.91 |
| Maiden 22 °C | April 2008 | 2.28 | 0.40 | 2.32 | 0.58 |
| Repeat 22 °C | April 2008 | 3.45 | 0.86 | 4.07 | 1.30 |
