# Supplementary material for: Hepatic reference gene selection in adult and juvenile female Atlantic salmon at normal and elevated temperatures
Source: BMC Res Notes. 2012 Jan 10;5:21. doi: 10.1186/1756-0500-5-21 (PMC3392733; doi:10.1186/1756-0500-5-21)
Supplement: Addtional file 3 — Table S3. Abundance of hepatic mRNA transcripts for Tbp, Hprt1 and Ef1α in juvenile Atlantic salmon given a blank or E2 silastic implant at 14 or 22°C. [file 1756-0500-5-21-S3.DOCX]

**Table 3 Abundance of hepatic mRNA transcripts for Tbp, Hprt1 and Ef1α in juvenile Atlantic salmon given a blank or E_2_ silastic implant at 14 or 22 °C**

| **Experimental group** | **Day** | **Mean candidate reference gene C_q_ and SEM** | | | | | |
| --- | --- | --- | --- | --- | --- | --- | --- |
|  |  | **Tbp** | | **Hprt1** | | **Ef1α** | |
| E_2_ 14 °C | 3 | 26.47 | 0.95 | 19.73 | 0.63 | 14.43 | 0.39 |
| Blank 14 °C | 3 | 27.67 | 0.75 | 19.93 | 0.89 | 14.13 | 0.33 |
| E_2_ 22 °C | 3 | 26.71 | 0.45 | 20.82 | 0.17 | 14.82 | 0.17 |
| Blank 22 °C | 3 | 27.15 | 0.51 | 21.22 | 0.56 | 14.06 | 0.36 |
| E_2_ 14 °C | 7 | 26.51 | 0.49 | 20.63 | 0.36 | 14.94 | 0.35 |
| Blank 14 °C | 7 | 28.09 | 0.23 | 20.74 | 0.16 | 14.98 | 0.09 |
| E_2_ 22 °C | 7 | 27.85 | 0.12 | 21.26 | 0.23 | 14.92 | 0.36 |
| Blank 22 °C | 7 | 26.82 | 0.26 | 20.62 | 0.34 | 14.23 | 0.25 |
| E_2_ 14 °C | 14 | 26.88 | 0.34 | 20.87 | 0.31 | 14.97 | 0.24 |
| Blank 14 °C | 14 | 26.82 | 0.31 | 20.42 | 0.26 | 14.91 | 0.27 |
| E_2_ 22 °C | 14 | 27.52 | 0.64 | 21.89 | 0.58 | 14.85 | 0.15 |
| Blank 22 °C | 14 | 28.52 | 0.31 | 21.17 | 0.17 | 14.84 | 0.23 |
